# Pre-crastination and path planning: Evidence for cognitive frontloading, a new sibling for cognitive offloading

**DOI:** 10.1007/s00426-025-02199-w

**Published:** 2025-11-13

**Authors:** Sophia Angleton, Simran Bhatia, Arineh Moradian, David A. Rosenbaum

**Affiliations:** 1 Department of Psychology, University of Oregon, Eugene, OR 97403 USA; 2 Department of Psychology, University of California,, 900 University Ave., Riverside, Riverside, CA 92521 USA

## Abstract

Pre-crastination is the tendency to hasten task completion, even at the expense of extra effort. Discovered in 2014, it is a widespread phenomenon hypothesized to reduce cognitive effort. We sought to determine whether pre-crastination holds for multi-step path planning. In Experiments 1 and 2, our university-student participants saw all but one of the numbers from 1 to 6 on a computer screen and, when ready, hit the spacebar (Experiment 1) or touched the trackpad (Experiment 2) to reveal the missing number. In both experiments, they then clicked on the targets sequentially as quickly as possible. The time for the first target was longer than for any other target even when noninitial targets were withheld from the preview. A third experiment confirmed that the lengthening of the first response was due to resolution of response uncertainty. The results as a whole confirmed that participants hastened task completion by expending extra effort up front (spending extra time to set up all the responses they would perform). The study extends the reach of pre-crastination and points to the general tendency, now manifest in a growing number of contexts, that, when possible, people complete decision-making about forthcoming response sequences as soon as they can. We refer to this tendency as cognitive frontloading and offer it as a new companion to cognitive offloading, which has been much studied. Both methods reduce memory demands, but in different ways.

## Introduction

In everyday life, plans for intended actions must often be formed without complete knowledge of all the actions that will be necessary. Little is known about how such plans are formed and implemented. We sought to shed light on this topic, motivated largely by our interest in pre-crastination, a phenomenon discovered by Rosenbaum et al. (2014) and defined by them as the tendency to complete, or at least begin, tasks as soon as possible, even at the expense of extra physical effort. Since then, the term has come to be used less restrictively, without reference to physical effort, per se. We follow that less restrictive convention here. Meanwhile, we adhere to a practice adopted by Rosenbaum et al. (2014) to use the hyphen in “pre-crastinate” to reduce the chance that the word is misread as “procrastination,” its antonym.

The task in which pre-crastination was first demonstrated was pursued in experiments on cognition and biomechanics. The question was how people would judge the costs of carrying light objects over long distances versus heavy objects over shorter distances. How would people trade off weight and distance?

Prior to doing a direct test of the alternative hypotheses that bore on this question, Rosenbaum et al. (2014) checked their procedure with equal-weight objects. They asked university students to do whatever the students found easier: (a) walk a short distance to pick up an object and carry it to a far destination; or (b) walk a longer distance to pick up another object and carry it a shorter distance to the same final destination. Surprisingly, most participants chose the closer object even though it had to be carried further. The same tendency was observed in other studies (David et al., 2024; Ma & Zhang, 2023; Patterson & Kahan, 2019; Rosenbaum & Dettling, 2023; VonderHaar et al., 2019). Analogous task preferences were found in operant conditioning experiments with nonhuman subjects (Rayburn-Reeves et al., 2011; Wasserman & Brzykcy, 2015; Wasserman, 2019).

In summarizing this tendency, Rosenbaum et al. (2014) coined the word pre-crastination to echo what the (human) participants said they were doing – trying to get the task done more quickly. In fact, there was no difference in task-completion times for the near-bucket and far-bucket methods, as later confirmed by Rosenbaum and Sauerberger (2019). The interpretation given by Rosenbaum et al. (2014) to their participants’ reports was that completing the subgoal earlier helped participants feel like they were nearing the main goal sooner rather than later. Wanting to get things done as soon as possible is, of course, the opposite of procrastination. The term pre-crastination was coined by Rosenbaum et al. (2014) to capture this difference.

The most obvious explanation for pre-crastination is impulsiveness: The near object attracts attention and comprises “low-hanging fruit.” Many studies have ruled out this possibility. For example, in one experiment, Rosenbaum et al. (2014) showed that the near-bucket preference persisted even when participants had to wait up to 4 s before leaving the start line. The signal to leave the start line was displayed on a screen at the end of the walkway, which meant that participants had to look down the walkway for the full 4 s and could not just glance at whatever was closest to them and head for whatever object happened to catch their attention. Consistent with this conclusion, other studies have shown, using personality inventories, that impulsiveness scores are uncorrelated with the likelihood of pre-crastinating (choosing the near bucket when it must be carried farther than the other bucket). Rather than being a tendency to avoid impulsiveness, the tendency to pre-crastinate has actually been identified as a new, distinct trait (Gehrig et al., 2023). For reviews, see Rosenbaum et al. (2019) and Rosenbaum and Sauerberger (2022).

The explanation for pre-crastination that is most promising is that it stems from the desire to clear the mind of as-yet-uncompleted goals (Rosenbaum et al., 2014, 2019; Rosenbaum & Sauerberger, 2022). Consistent with this view, Fournier et al. (2018) showed that the likelihood of pre-crastination increased with an extra memory load. In the procedure of Fournier et al. (2018), participants were asked to walk down an alley to return with two buckets that stood before them, one close and other farther away. Participants were asked to bring back both buckets in one trip. The question was which of two strategies they would follow: (1) Would they walk around the close bucket, pick up the far bucket, and then pick up the close bucket on the way back; or (2) Would they pick up the close bucket on the way out, carry it with them while picking up the far bucket, and then return with both buckets? If the latter strategy were used, it could be viewed as an instance of pre-crastination.

Surprisingly, most participants used the pre-crastination strategy. Most participants picked up the near bucket on the way out, then picked up the far bucket, and then returned with both. Furthermore, consistent with the hypothesis that pre-crastination reduces memory load, the tendency to pick up the near bucket on the way out was stronger when participants performed the task with a memory load (memorizing a list before traversing the alley and recalling the list upon returning). Other experiments using the same general logic further supported the memory-load-reduction hypothesis (David et al., 2024; Ma & Zhang, 2023; Patterson & Kahan, 2019; Rosenbaum & Dettling, 2023), or CLEAR hypothesis, as VonderHaar et al. (2019) called it.

Using a very different procedure that was designed, nonetheless, to test the same idea, Rosenbaum et al. (2022) measured reaction times to see whether people would be predisposed to make decisions as soon as possible even when there was no reward for doing so. In these experiments, participants were required to make speeded yes-or-no judgments twice, rather than once, per trial.^1^ Participants in the double-response task were told that the only outcome that mattered was the accuracy of the second response. Rosenbaum et al. (2022) found that the second responses almost always matched the first responses, and the first responses had much longer latencies than did the second responses. Rosenbaum et al. (2022) suggested that participants sought to complete decision-making as soon as possible, consistent with pre-crastination.

**Fig. 1:**
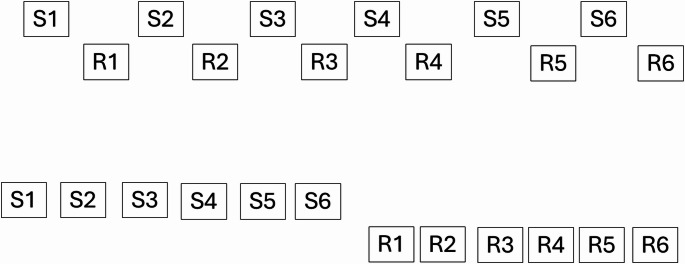
Chaining and frontloading. Top panel: Chaining (alternating between processing stimuli 1–6 and generating the associated responses); Bottom panel: Frontloading (processing all stimuli and then generating all their responses). Two rows are shown per panel to refer to a stimulus-processing channel (above) and a response-processing channel (below). Frontloading delays the start of responding but allows successive responses to be made rapidly. Whether chaining or frontloading is used depends on the storage capacity of the relevant stimulus and response stores. If the storage capacity is 1, then chaining is the only option, and the term frontloading carries no weight. In the present experiments, we tested for frontloading given a presumed storage capacity of 6 items

In the present study, we set out to determine whether the same tendency holds in a domain where it has not yet been tested and where, a priori, there was no reason to expect it to apply. The domain was path planning. For the task we studied, participants could either start on a path and resolve a point of uncertainty once they reached the uncertain point, or they could resolve the uncertainty up front. We sought to address this question to test for a phenomenon we call cognitive frontloading, which we define as the use of information to prepare all responses for an upcoming response sequence as soon as possible, as illustrated in the bottom panel of Fig. 1.

Cognitive frontloading can be contrasted with chaining, where responses are generated immediately after their respective stimuli are processed (Fig. 1 top panel). The term cognitive frontloading can be viewed as a new “sibling” for cognitive offloading, which has been studied extensively. The connections are discussed near the end of the article.

## Experiment 1

### Overview of task and models

**Fig. 2:**
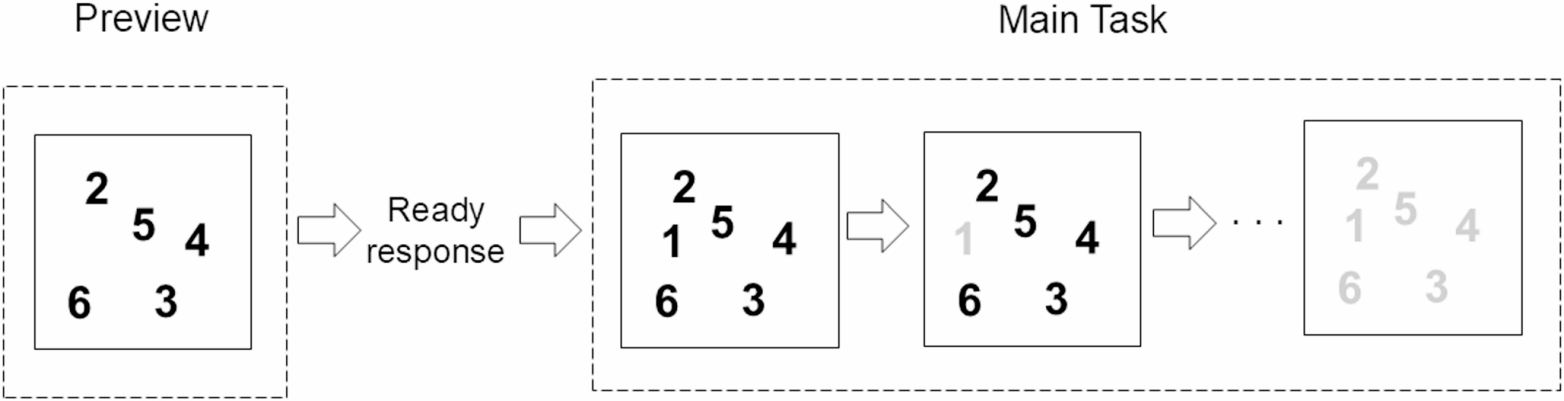
Events in a typical trial. The preview showed all but one of the number targets. The participant made a ready response (pressing the spacebar in Experiment 1) which caused the computer to add the initially missing number. The participant then clicked on targets 1–6. Each number turned gray if it was clicked close enough to the next one due. The first panel within “Main Task” shows the display after the ready response (spacebar press) was made; the 1 was added in this case. The second panel within “Main Task” shows the effect of clicking close enough to 1 when it was due. Finally (last panel within “Main Task”), all six targets have been greyed out. Feedback about total time for the main task, including whether the total time was the shortest so far, was given after the responses were done (not shown here). The aiming task was quite easy. A number was considered clicked-on if the clicking occurred anywhere in a circle centered on the number; the circle’s radius was half the minimum possible distance between the numbers. In this example, the numbers 1 and 5 depict the minimum vertical distance between targets, as do the numbers 5 and 2. Similarly, the numbers 1 and 2 depict the minimum horizontal distance between targets, as do the numbers 2 and 5. Each display filled the laptop screen

An overview of the procedure will help clarify the models of interest. In each trial, our university-student participants saw all but one of the integers 1 through 6 on a computer screen (Fig. 2). The numbers were arranged unpredictably from trial to trial. Participants could look at the numbers and, when ready, hit the spacebar. This caused the computer to add the previously missing number to the display. At this time, participants could use the trackpad to bring a cursor to each of the numbers and click on or near it (see below). The numbers were to be clicked sequentially (1 to 6) so as to minimize the time between the spacebar press and the final target click. When the next required number was clicked, it turned gray. After the last target was clicked, participants got feedback about the time between the spacebar press and the click on the final target.

The main independent variable was the serial position of the missing target. The main dependent variable was the time per target. Participants were told to take as long as needed on the preview and that this time would not affect their scores, which were based solely on the target completion time. Participants were told as well (and quickly found out in the opening trials) that a click was not registered (a target did not turn gray) if the click was on a target that was out of order (e.g., 3 before 2) or if the click was too far from any target (see below). The feedback included a congratulatory message if the total time after termination of the preview was the lowest so far.

Four models were of special interest. All were juxtaposed to the null model, according to which there would be no systematic effect of the independent variable (missing target number) on the target acquisition times. The four models differed on two dimensions – whether preparing the initially uncertain target occurred as late as possible (*procrastination*) or as early as possible (*pre-crastination*), and whether the targeting task as a whole could be completed quickly or not. We called the four models Slow Procrastination, Fast Procrastination, Slow Pre-crastination, and Fast Pre-crastination. The claims of the models are spelled out below after we orient the reader to Table 1, where the model’s predictions are laid out.

**Table 1:** Ordinal levels of time (1 lowest up to 3) for each response depending on which response was missing from the preview, according to four models of interest

Model 1: Slow Procrastination Response						
Missing	1	2	3	4	5	6
1	3	3	3	3	3	3
2	1	3	3	3	3	3
3	1	1	3	3	3	3
4	1	1	1	3	3	3
5	1	1	1	1	3	3
6	1	1	1	1	1	3
Model 2: Fast Procrastination Response						
Missing	1	2	3	4	5	6
1	3	1	1	1	1	1
2	1	3	1	1	1	1
3	1	1	3	1	1	1
4	1	1	1	3	1	1
5	1	1	1	1	3	1
6	1	1	1	1	1	3
Model 3: Slow Pre-crastination Response						
Missing	1	2	3	4	5	6
1	3	1	1	1	1	1
2	3	1	1	1	1	1
3	3	1	1	1	1	1
4	3	1	1	1	1	1
5	3	1	1	1	1	1
6	3	1	1	1	1	1
Model 4: Fast Pre-crastination Response						
Missing	1	2	3	4	5	6
1	3	1	1	1	1	1
2	2	1	1	1	1	1
3	2	1	1	1	1	1
4	2	1	1	1	1	1
5	2	1	1	1	1	1
6	2	1	1	1	1	1

In Table 1, the columns are response number (target 1 through 6), while the rows are the missing target numbers (which of the six targets was withheld from the preview). The cells of the table have three time levels: 1, 2, and 3. These correspond to the time to execute a response that was fully prepared (time level = 1), the longer time to execute and, before that, complete preparation of a response that had been partly but not fully prepared (time level = 2), and the still longer time to execute and, before that, complete preparation of a response that had not prepared at all (time level = 3).

According to Model 1, the Slow Procrastination model, participants would use the preview period to specify responses for the initially certain responses up to but not beyond the initially uncertain target. When the preview period was over, participants would generate the specified responses and then successively specify and execute each of the remaining responses. The mean time to complete the tasks would be 13 steps, that is, (18 + 16 + 14 + 12 + 10 + 8)/6.^2^

According to Model 2, the Fast Procrastination model, participants would use the preview period to specify responses for all of the initially certain targets, including those that would occur after the initially uncertain target. When the preview period was over, participants would execute the specified responses and then specify and execute the initially uncertain response. After that, all of the remaining responses would be executed without the need to specify them. The mean time to complete the tasks would be 8 steps, that is (8 × 6)/6.

According to Model 3, the Slow Pre-crastination model, participants would use the preview period to specify responses for all of the initially certain targets and, when the preview period was over, would specify any initially uncertain response before the first response was executed. All subsequent responses would then be executed. The mean time to complete the tasks would correspond to 8 time steps (the same as for the Fast Procrastination model).

Finally, according to Model 4, the Fast Pre-crastination model, participants would use the preview period to fully specify responses for all of the initially certain targets (as in Models 2 and 3), but with the following provisos. When the preview period was over, if the first response was initially uncertain, participants would specify that response and then execute it, and all remaining responses would be executed after that. By contrast, when the preview period was over and the initially uncertain response was *not* the first response, participants would begin the process of specifying the previously uncertain response and then, while executing the first response, complete the specification of the initially uncertain response, after which all remaining responses would be executed. This method would enable faster first responses on average than with the Slow Pre-crastination model (but not faster first responses on average than with the Fast Procrastination model or Slow Procrastination model). The Fast Pre-crastination model would yield the shortest mean task completion times (7.17 time steps).^3^ In addition, it could yield shorter preview times when the first response was initially uncertain than when the first response was initially certain because, when the first response was initially uncertain, none of its preparation could occur in the preview period, but later responses (all certain) could be prepared either fully or partially in the preview period, with their preparation being completed just after the preview period in the latter case. By contrast, when the first response was initially certain, it would have to be prepared fully in the preview period to avoid an exceptionally long first-response time. The luxury of completing response preparation just after the preview period would not apply when the first response was initially certain.

### Method

The experiment was controlled with a MATLAB program written by the authors and available upon request. The numbers were displayed in black Arial font (size 14) at random locations per trial except for the constraint that no two numbers could be less than 10% apart relative to the height or width of the square figure window. A cursor click counted as correct if it fell within 10% of the spatial zone centered on the needed target. If the clicked target was correct, it turned grey and the time was registered; otherwise, it did not change color and the time was not registered; this signaled the need for another click for the target that was due. We did not record clicks on wrong targets or clicks outside the catchment area of a target. Participants were tested in 60 trials (each of the 6 numbers omitted from the preview 10 times in random order).

To arrive at the required number of participants, we assumed a moderate effect size (*d* = 0.5). Via an a priori power analysis conducted with G*Power 3 (Faul et al., 2007), we found that a sample size of 34 participants would suffice to detect a one-sample effect on times for initially certain versus initially uncertain responses (*a* = 0.05, 80% statistical power). Owing to the availability of extra students who wished to do the study, we tested 40 subjects. All were undergraduates who participated for UCR Psychology Department course credit. The experiment was approved by the UCR Institutional Review Board. All participants were treated in accordance with ethical guidelines.

### Results

The results to be presented concern the six click times in each of the conditions of the experiment – when the first response was initially uncertain, when the second response was initially uncertain, and so on, up to the sixth. The data to be considered come from trials 7–60. The first six trials were considered practice.

Preliminary analysis indicated that the mean times, T1-T6, for responses 1 through 6, respectively, were unduly affected by a small number of extreme outliers, with the vast majority of times (> 99% observations) having values of 3 s or less. We only included the latter times in our further analysis, though the patterns to be reported were consistent with all of the data included. The correlation between the two data sets (with and without filtering) was *r* = .99934, Fisher’s *z* = 1.946, *p* = 2 × 10^−10^. All of the data from this experiment and the next ones are available at https://osf.io/uphfj/.

**Fig. 3:**
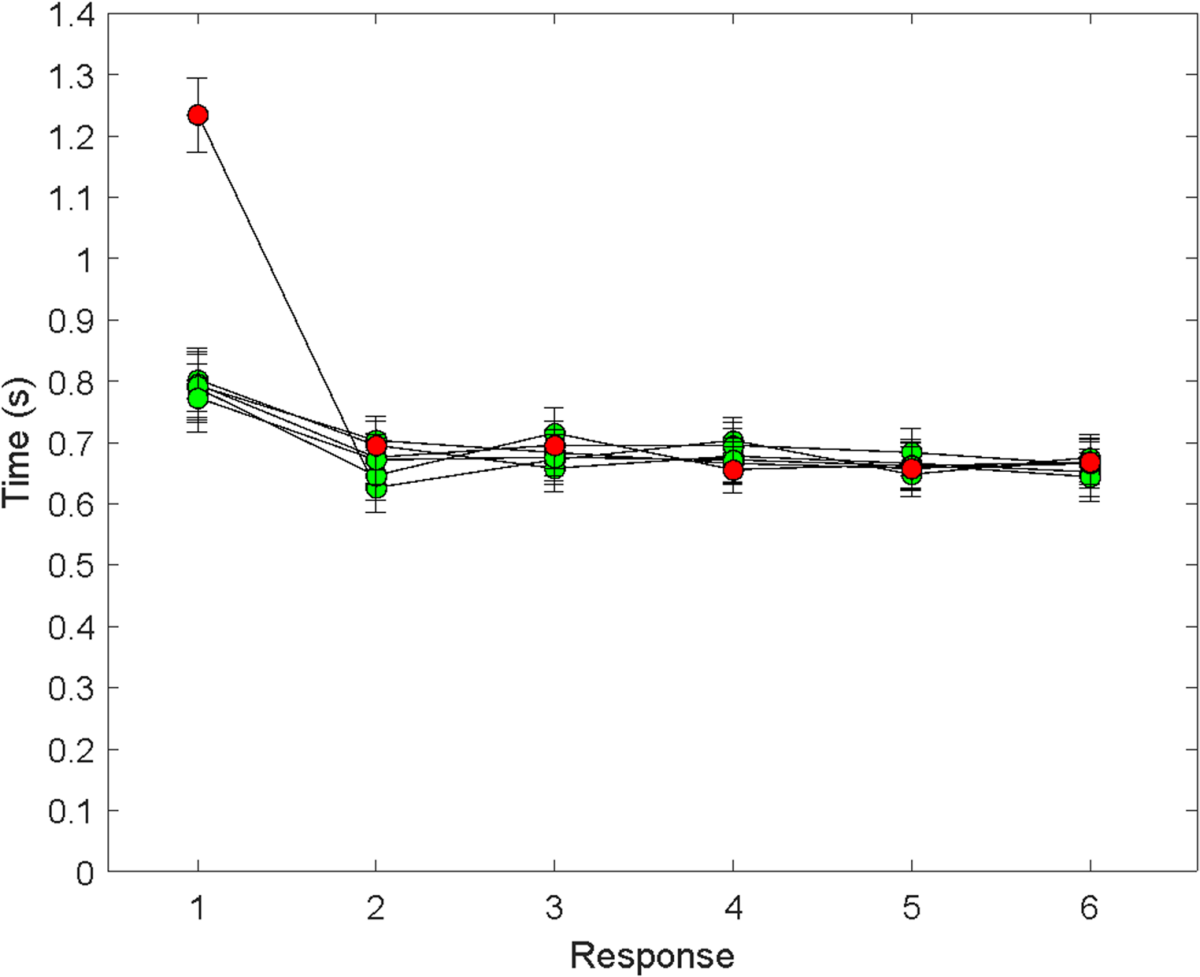
Mean response time (± 1 SE) as a function of response number in Experiment 1 (trials 7–60). Each curve corresponds to a sequence in which Target 1, 2, 3, 4, 5, or 6 was withheld from the preview. Red points are for targets that were withheld from the preview. Green points are for targets that were shown in the preview. The scale of the *y* axis was chosen to be the same for this and the subsequent data graphs

Figure 3 shows the means and standard errors for the times to click on targets 1 through 6 in each of the six uncertainty conditions. As seen in the figure, T1 was longer than T2 through T6. For T1, there was a massive effect of which response was initially uncertain. Consistent with the Fast Pre-crastination model, T1 was longer when target 1 was initially uncertain (*M* = 1.23 s, *SE* = 0.06 s) than when target 1 was initially certain (*M* = 0.79 s, *SE* = 0.05 s), paired*t* (one-tailed) = 10.47,*df* = 39, *p* <.001, Cohen’s effect size score for repeated-measures (recommended by Lakens, 2013), *d*_*rm*_ = 1.66. In addition, T1 when target 1 was initially uncertain was longer than the mean of T2 through T6 (*M* = 0.67 s, *SE* = 0.04 s), paired *t* (one-tailed) = 13.68, *df* = 39, *p* <.001, *d*_*rm*_ = 2.16. Finally, T1 when target 1 was initially certain was longer than the mean of T2-T6, paired *t* (one-tailed) = 3.75, *df* = 39, *p* <.001, *d*_*rm*_ = 0.59.

Each of the four models was fit to each subject’s data. We sought parameter estimates for the models that had ordinal relations prescribed by the model (Table 1). The parameter estimates we adopted were obtained through Monte Carlo simulation and were chosen to minimize the sum of squared differences between the 36 mean observed times and the predicted times per subject.

Using adjusted R^2^ values to penalize the model with one more parameter than the others (the Fast Pre-crastination model), we found that the Fast Pre-crastination model yielded the largest adjusted R^2^ value for 37 out of 40 participants. The probability of this event was vanishingly small: *p* = 2.2066e-^19^. For the three participants who did not have Fast Pre-crastination in the top spot, their mean adjusted R^2^ values (and standard errors) for the Fast Pre-crastination model and Slow Pre-crastination model were 0.430 (0.035) and 0.443 (0.222), respectively. Over all 40 subjects (so including the three just mentioned), the mean adjusted R^2^ values (and standard errors) were 0.555 (0.035) and 0.331 (0.045), for the Fast Pre-crastination and Slow Pre-crastination models, respectively. The mean adjusted R^2^ values for the Fast Procrastination and Slow Procrastination models were essentially zero: 0.011 and 0.036, respectively.

### Discussion

In Experiment 1, we asked how participants would carry out sequential aiming tasks when all but one of the targets was shown in advance. We found that participants took longer for first targets than later targets, especially when the first target was initially uncertain. The results were inconsistent with both of the procrastination models but were consistent with the pre-crastination models. The Fast Pre-crastination did better than the Slow Pre-crastination model for the vast majority of participants. Participants hastened task completion by spending extra time at the starts of the response production phase and in a way that was highly adaptive insofar as it was tuned to the serial position of the initially missing target.

## Experiment 2

There was a reason to be cautions about the interpretation of the Experiment 1 results. According to an alternative account, a possible artifact may have influenced the times in the first experiment. The possible artifact was related to the fact that for first-target responses, participants moved their hands from the space bar to the trackpad whereas for later-target responses, participants moved their hands from place to place on the trackpad. Conceivably, the former movements took longer than the latter, and even if the sheer movement time was not much different – we know of no specific study that directly answers this question – there may have been a switch cost associated with the shift from one mode of responding to another. If either or both of these hypotheses were true, one could question the main theoretical conclusion of Experiment 1, namely, that preparation of non-initial responses occurred before completion of the second response. Instead, it is possible that there was just one condition in which response uncertainty cost extra time, the one in which the first target was uncertain.

### Method

To test the alternative account in Experiment 2, we asked participants to terminate the preview by moving a cursor with the trackpad and clicking on the trackpad to indicate where they thought the missing target would be. Clicking the screen this way had the same effect as pressing the spacebar in Experiment 1. It caused the missing target to appear, at which point participants moved the cursor via the trackpad and clicked on the targets, as they had in Experiment 1.

Participants in Experiment 2 were told that their preview time would not affect their scores (as in Experiment 1) and that their prediction accuracy would not affect their scores either. The main instruction was to click the targets sequentially as quickly as possible after the preview period ended (when the initially missing target was added to the display). In all other respects, the method was the same as in Experiment 1. Thirty-eight new UCR students participated. As in Experiment 1, the number of participants tested was more than the number of participants specified through our initial power analysis (34), but we welcomed the four additional students. All of the participants took part under the same auspices as in Experiment 1.

### Results

As in the data analysis for Experiment 1, the results to be considered excluded the first six trials, and as in Experiment 1, we checked for outliers in the response-time distribution to avoid undue influences from extreme scores. We found that with the same cutoff as in Experiment 1 (3 s), only a small number of observations exceeded this value. The remaining > 99% of the data were entered into the analyses. We only included the latter times in our further analysis, though the patterns to be reported were consistent with all of the data included. The correlation between the two data sets (with and without filtering) was *r* =.83, Fisher’s*z* = 1.168, *p* = 1.99e-11.

**Fig. 4:**
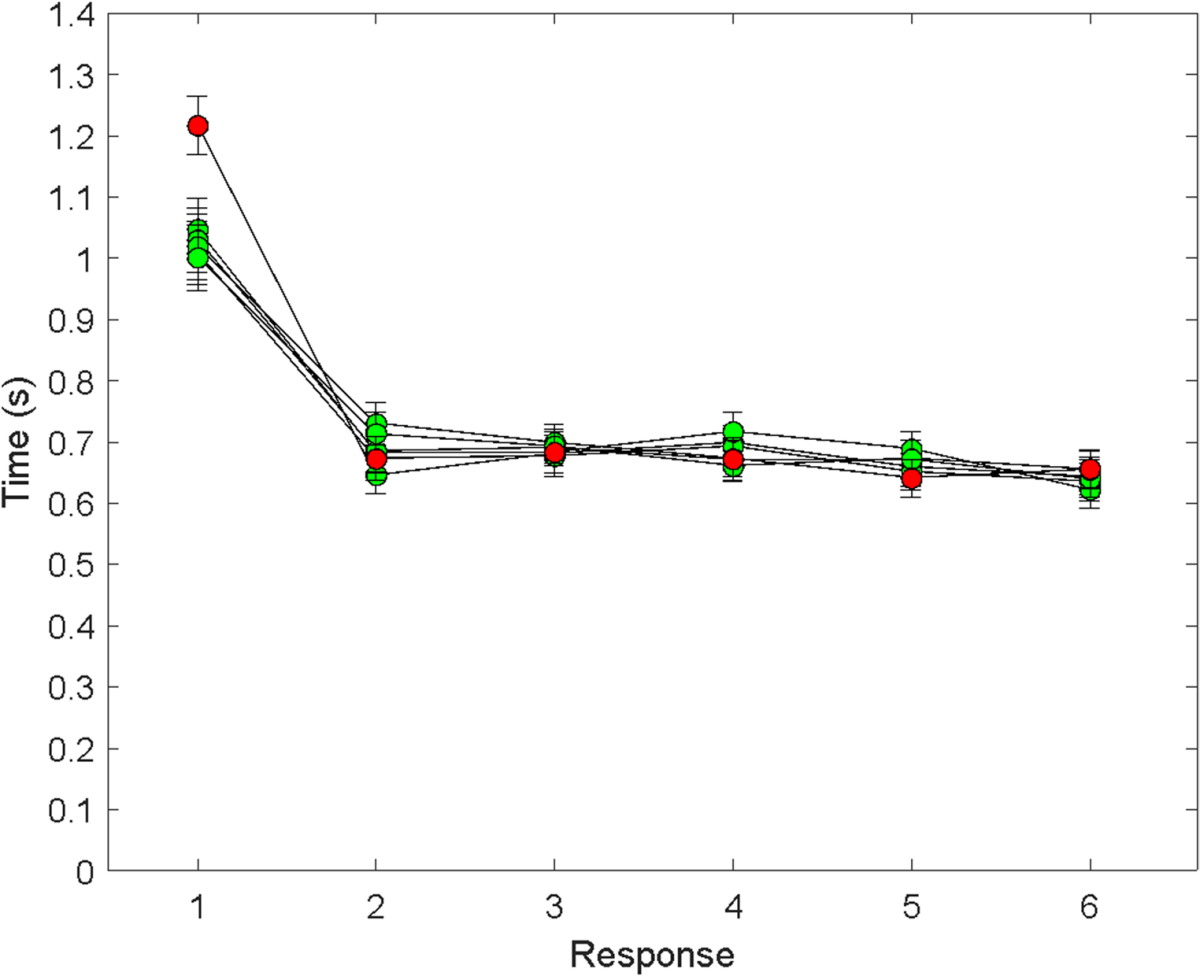
Mean response time (± 1 SE) as a function of response number in Experiment 2 (trials 7–60). Symbol conventions the same as before. The scale of the *y* axis was chosen to be the same for all of the data graphs

Figure 4 shows the mean times just referred to for the filtered data for the first through sixth responses when the first through sixth targets were initially uncertain. The pattern was similar to the one in Experiment 1. The only noticeable difference was that when the second through sixth responses were initially uncertain, T1 was longer than in Experiment 1. The other times – namely, T1 when target 1 was initially certain, and the subsequent times, T2-T6 – were remarkably similar to their counterparts in the first experiment.

The data were analyzed in the same manner as the data in Experiment 1. Consistent with the Fast Pre-crastination model, T1 was longer when target 1 was initially uncertain (*M* = 1.22 s, *SE* = 0.05 s) than when target 1 was initially certain (*M* = 1.02 s, *SE* = 0.05 s), paired *t* (one-tailed) = 5.66, *df* = 37, *p* <.001, *d*_*rm*_ = 0.90. In addition, T1 when target 1 was initially uncertain was longer than the mean of T2 through T6 (*M* = 0.67 s, *SE* = 0.03 s), paired *t* (one-tailed) = 14.47, *df* = 37, *p* <.001, *d*_*rm*_ = 2.35. Finally, T1 when target 1 was initially certain (*M* = 1.02 s, *SE* = 0.05 s) was longer than the mean of T2-T6 (0.67 s, *SE* = 0.03 s), paired *t* (one-tailed) = 8.01, *df* = 37, *p* <.001, *d*_*rm*_ = 1.30.

As in Experiment 1, each of the four models was fitted to each subject’s data, with the same aims and constraints as in the first experiment. Again, using adjusted R^2^ values to penalize the model with one more parameter (the Fast Pre-crastination model), we found that that model yielded the largest adjusted R^2^ value for 28 of the 38 participants. The probability of this event was tiny, *p* = 3.69e-^10^. For the ten participants who did not have Fast Pre-crastination in the top spot, their mean adjusted R^2^ values (and standard errors) for the Fast Pre-crastination and Slow Pre-crastination models were 0.661 (0.08) and 0.674 (0.08), respectively. Over all 38 subjects, the mean adjusted R^2^ values (and standard errors) were 0.683 (0.04) and 0.629 (0.04), for the Fast Pre-crastination and Slow Pre-crastination models, respectively. The mean adjusted R^2^ values for the Slow Procrastination and Fast Procrastination models were close to zero: 0.001 and 0.002, respectively.

### Discussion

The second experiment was designed to test the hypothesis that an artifact may have affected the results of Experiment 1. Because participants in Experiment 1 moved their hands from the space bar to the trackpad for the first target but moved their hands from place to place on the trackpad for the later targets, it was possible that this physical difference and/or task switching elevated the first-target times (T1) such that the only condition that took longer because of the need to resolve response uncertainty was the one in which target 1 was initially uncertain. If that were the case, one could say that cognitive frontloading was not really demonstrated in Experiment 1.

We were able to dispel this possibility in Experiment 2 by replacing the spacebar response with the trackpad response. Via this change in procedure, we found that the change did not eliminate T1 lengthening. Because the results of Experiment 2 were very similar to the results of Experiment 1, we infer that the Experiment 2 results further support the Fast Pre-crastination model.

As for why T1 was longer in Experiment 2 than in Experiment 1 when the second through sixth responses were initially uncertain, we surmise that in Experiment 2 participants switched from attending to a predicted target location to attending to an actual target location before making a response. In Experiment 2, participants were asked to predict where the missing target would be, while in Experiment 1 they were not. Requesting the prediction may have led to a stronger desire to determine where the initially missing target was before making any responses. We view this as an issue for future research, not as one that needs to be resolved to reach a general conclusion about the results as a whole, namely that the Fast Pre-crastination model provided the best account of the target click times for the two experiments. As in Experiment 1, participants hastened task completion by expending effort at the starts of the response production phase (spending extra time to specify all of the upcoming responses).

## Experiment 3

The final experiment provided a check of the claim that resolving response uncertainty explained the finding that times were longer for the first response than for the later responses. Conceivably, some other factor caused this result, such as switching from a preview mode to an action mode or becoming aroused to enable rapid responding.

To test that some other factor caused the T1 lengthening, we conducted a third experiment in which there was no uncertainty about any of the targets. Participants were shown previews of all six targets in all of the trials. As in Experiment 1, participants signaled when they were finished previewing by pressing the spacebar, after which they used the touchpad to click on the targets as quickly as possible, as in the first two experiments.

We reasoned that if T1 lengthening in Experiments 1 and 2 reflected planning (resolving response uncertainty) rather than some other process like switching from a preview mode to an action mode or becoming generally aroused, we would not see T1 lengthening as we had before. On the other hand, if T1 lengthening in Experiments 1 and 2 reflected some process other than planning, we would see T1 lengthening as we had before.

### Method

To test the alternative account in Experiment 3, we previewed all the targets in every trial. Participants were told to terminate the preview with the spacebar and, after that, to click on the targets in succession, minimizing the time until completion of the entire 6-response sequence. As in the first two experiments, we said nothing about minimizing the time for any particular target. Thirty-five UCR undergraduates participated for academic credit. None had been in the other experiments. The design and procedure were otherwise the same as in Experiment 1.

### Results

**Fig. 5:**
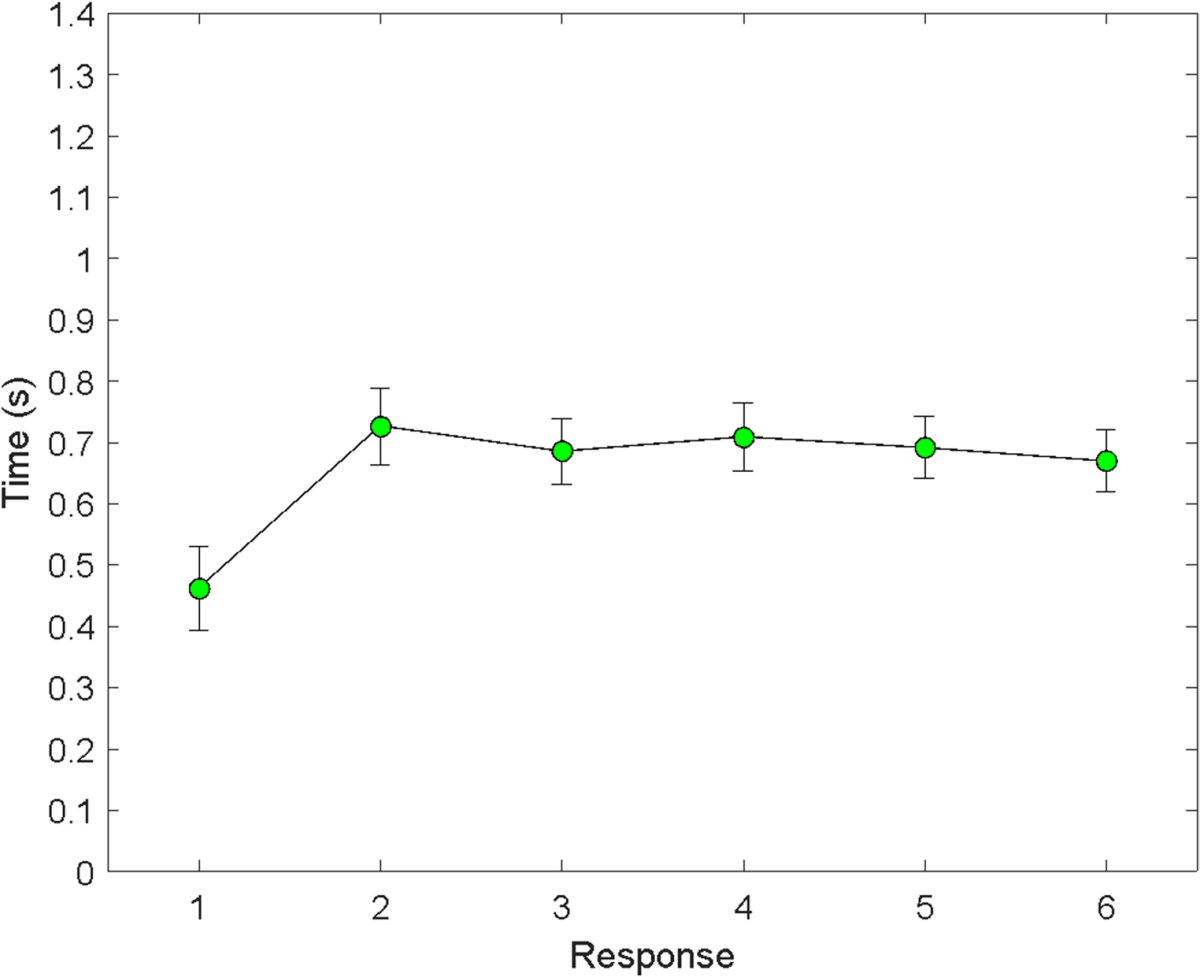
Mean response time (± 1 SE) as a function of response number in Experiment 3 (trials 7–60). Only green dots are used here because all of the targets were previewed. The scale for the *y* axis is the same as for Experiments 1 and 2

For consistency with the analyses of Experiment 1, we limited the target times to 3 s or less, which kept 99.4% of the trials. The results were virtually the same if outliers were kept or not. The correlation between the two data sets (with and without filtering) was *r* =.99,934, Fisher’s *z* = 4.008, *p* = 2 × 10^−10^. The filtered times are shown in Fig. 5, where it is seen that T1 was not longer than subsequent times; in fact, it was actually shorter than the subsequent times.

We conducted a repeated-measures ANOVA to evaluate the effect of target number on times T1 through T6. The ANOVA yielded an effect of response number that was unlikely to have arisen by chance alone if the null hypothesis were true, *F*(5,170) = 15.78, *p* = 9.4274e-13. Pairwise comparisons (Bonferroni-corrected) indicated that T1 was different from every other time. The largest *p* value for these six comparisons was *p* =.0088145. For the other pairwise comparisons, T2 vs. T3 had a *p* value of 0.041392, but all other comparisons equaled or exceeded *p* =.2959. A paired *t* test (one-tailed) designed to test the hypothesis that T1 was less than the mean of T2-T6 yielded, *t*(34) = −4.28, *p* =.0001, *d*_*rm*_ = 0.4497.

### Discussion

The third experiment was designed to check whether the lengthening of T1 observed in the first two experiments was actually due to specification of the originally unpreviewed target. The experiment was designed to test the alternative possibility that T1 lengthening in Experiments 1 and 2 had some source not based on planning per se, such as taking time to switch from a preview mode to an action mode or increasing arousal to enable fast, spatially directed responses.

To distinguish between these possibilities, we showed all the targets in advance, reasoning that if the previously observed T1 lengthening was due to planning for the target that was originally withheld, then that T1 lengthening would not be seen when all targets were previewed. Conversely, if the previously observed T1 lengthening was *not* due to planning for the target that was originally withheld, then T1 would still be observed.

The results corroborated the planning prediction. T1 was not longer than the subsequent target times. In fact, T1 was shorter than the subsequent times. This result was not expected but, in retrospect, was consistent with the view that participants sought to minimize their total targeting time. In this instance, they apparently did so by concentrating their most intense, fastest performance at the beginning, also consistent with pre-crastination. As for why the second response had somewhat longer times than later responses, we can only speculate. Perhaps it was that that it took a little extra time to change the physical posture of the eyes, head, and hand from the one used for highly prepared first-target responding to a posture that allowed for shifting from target to target.

Finding that T1 was not longer than subsequent times in this experiment helps put to rest a concern raised by one of the reviewers. According to the reviewer, in Experiments 1 and 2 (the only experiments reported in the version the reviewer saw because Experiment 3 came along in response to this and other reviewers’ concerns), T1 may have been longer than later times (times for subsequent targets) because the numeral “1” was potentially harder to see than the other numerals. This could have been the case because “1” was narrower than the other numerals given the Arial 14-point font used for the targets. The results of Experiment 3 argue against this hypothesis, however, because, in the third experiment, the time to get to the “1” was not longer than the times to get to the other targets. Instead, the time was shorter.

## General discussion

This study concerned resolution of uncertainty about upcoming behavior. We asked university students to aim for targets numbered 1–6. In Experiments 1 and 2, all but one of the numbers were shown in advance. When participants felt prepared to go on after viewing the preview, they hit the spacebar in Experiment 1 or touched the trackpad in Experiment 2. This action, regardless of the nature of the physical response, caused the missing number to appear and started the period in which participants were supposed to click on the targets as quickly as possible. In Experiment 3, the procedure was different. All of the numbers were shown in advance. Participants hit the spacebar when they were ready, as in Experiment 1, and clicked on targets as rapidly as possible with the aim (as in the first two experiments) of minimizing the time between ending the preview and clicking on target 6.

The results provided clear support for one of the four models we considered, namely, the Fast Pre-crastination model. In agreement with that model, participants behaved in a way that minimized the total targeting time. Achieving that goal came at some expense, namely delaying the start of the targeting sequence. Impressively, participants could delay the start to a lesser degree when a noninitial response was withheld from the preview than when an initial response was withheld from the preview. This selectivity may have been achieved by beginning the specification of the previously withheld noninitial response and completing that specification while the first response was being executed. Leaving that last point aside – it is, at best, speculation – the results as a whole are consistent with pre-crastination in that extra effort (extra time) was spent up front to promote faster completion of the action sequences as a whole.

In the remainder of this General Discussion section, we take up three remaining issues. The first concerns the way our participants used preview. The second concerns limitations of this study. The third concerns future work and broader implications, including the suggestion that the data reported here, along with other data sets in related studies, point to a newly identified tendency in perception, action, and cognition – what we call cognitive frontloading.

### Preview times

Regarding the use of previews, we inferred that participants paid attention to the previews given the systematic relation between the serial position of the withheld target and the subsequent response times in Experiments 1 and 2, as well as the great speed with which participants initiated their targeting responses when all of the targets were previewed in the third experiment. Participants spent quite a bit of time previewing: 3.12 s on average (*SD* = 1.97 s) in Experiment 1 (when they were given no specific instructions about what to do while previewing; 2.99 s on average (*SD* = 1.45 s) in Experiment 2 (when they were told to predict), and 4.08 s on average (*SD* = 3.28 s) in Experiment 3 (when they were shown all the targets in advance).

**Fig. 6:**
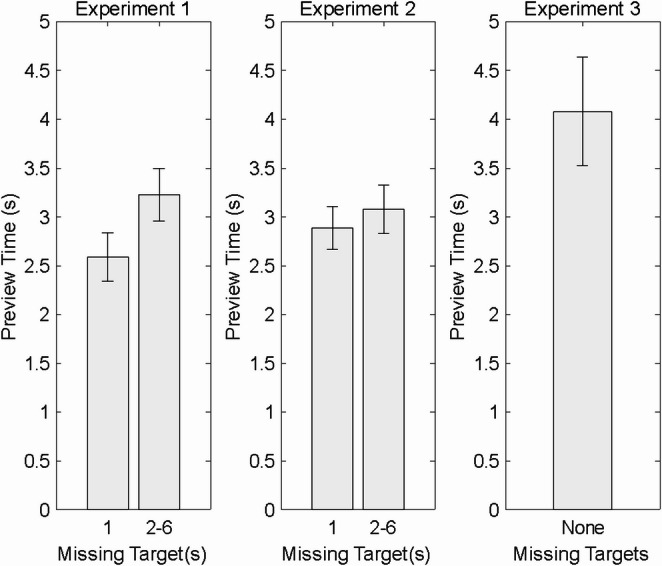
Mean preview time (± 1 SE) when target 1 was missing from the preview and when a later target was missing from the preview in Experiment 1 (left panel) and Experiment 2 (middle panel), and when no target was missing from the preview (right panel), which was always the case in Experiment 3

Consistent with the view that participants specified responses in advance when they could, we found, as shown in Fig. 6, that preview times were shorter when target 1 was missing from the preview than when targets 2–6 were missing from the preview (Fig. 6), both in Experiment 1 (one-tailed*t* = −4.96, *df* = 34,*p* <.001, *d*_*rm*_ = − 0.84) and in Experiment 2 (one-tailed*t* = −1.68, *df* = 37,*p* =.0509128017, *d*_*rm*_ = −0.27).^4^ Of course, no such comparison could be made in Experiment 3 because all of the targets were previewed in that experiment. Nonetheless, the preview time mean and standard error for Experiment 3 are included in Fig. 6 for visual comparison with the times mentioned in the last paragraph.

To assess the observed difference between the preview times for Experiment 3 and the preview times for Experiments 1 and 2, we conducted an independent-samples *t*-test comparing Experiment 3 (all targets shown) to the pooled data from Experiments 1 and 2 (one target withheld). The analysis showed that preview times were marginally significantly longer in Experiment 3 (*M* = 4.08 s, *SD* = 3.28) than in the combined Experiments 1 and 2 (*M* = 2.95 s, *SD* = 1.48), Welch’s *t* = 1.86, *df* = 47.1, *p* =.068. The effect size was moderate, *d*_*pooled*_ = 0.45. We used Welch’s t-test rather than Student’s t-test because the sample sizes of the groups being compared were unequal, as were the variances.

The result concerning shorter preview times when target 1 was missing goes against the Slow and Fast Procrastination models as well as the Slow Pre-crastination model. Regarding the Slow and Fast Procrastination models, if participants had adopted a procrastination strategy of either kind, they would have had no need to spend more time on preview displays that withheld noninitial targets. Seeing that a later target was withheld from the preview, they would have deferred specification of the associated response until later. Participants would have spent less time on previews without initial targets, not more time.

Regarding the Slow Pre-crastination model, had this model held, preview times would have been expected to be no different if initial or non-initial targets were withheld from the preview. In both cases, specification of the missing target would have occurred prior to completion of the first response and would have taken the same amount of T1 time, which is not what we observed.

Finally, the finding that shorter preview times were shorter when target 1 was missing accords with the Fast Pre-crastination model. When target 1 was missing from the preview, participants apparently appreciated that they would have to do all of their preparation for that target after the preview period was over, in which case they could terminate the preview sooner. By contrast, when a later target was missing from the preview, participants apparently appreciated that they could complete preparation of the later response before or during execution of the first response. They may have become even more prepared than usual to produce the first response, perhaps because preparing a later response at the same time would slow production of the first response. This interpretation would lead to the prediction that first response times could be shorter when there was no uncertainty about any targets than when there was uncertainty about one, which is just what we found in third experiment. Also consistent with this view, we found that preview times were longer in Experiment 3 than in Experiments 1 and 2, which accords with the hypothesis that the times that participants spent with the preview when all of the targets were in view may have included extra time for motor preparation per se (e.g., pre-activation of muscles).

### Limitations

This study had limitations. First, the measures we obtained were narrower than they could have been. We did not measure eye movements, for example, which could be a rich source of information about the use of previews and subsequent aiming. We also did not record the continuous position of the hand but instead recorded click locations. Another limitation concerned the click locations themselves. For the targeting task, we recorded when correct targets were clicked (when clicks were acceptably close to whichever target was next), but we did not record clicks at other locations, as noted earlier. Therefore, we do not know if and when clicks were made at wrong (out-of-sequence) targets or far from any target. Finally, we did not control or provide feedback about initial trackpad-to-cursor mappings. The cursor always began wherever it was last clicked. Greater control over this variable might be preferable in future studies. It might also be preferable to use a mouse rather than a trackpad (Warburton et al., 2025). Overcoming these limitations in future studies could provide more useful information.

### Broader implications

The present study has broader implications. The procedure used here sheds light on pre-crastination more generally and points to the possibility that pre-crastination reflects the broader tendency we have here named cognitive frontloading. Regarding pre-crastination, although pre-crastination was discovered in research on cognition and biomechanics, where it has continued to be explored (Rosenbaum & Dettling, 2023; Wood & Wong, 2024), it has been extended to studies of manual aiming (Blinch, & DeWinne, 2019), memory management (VonderHaar et al., 2019), individual differences (Fox et al., 2025; Fournier et al., 2025; Gehrig et al., 2023; Ma & Zhang, 2023; Wang & Sun, 2025), and joint action by pairs of performers (Karlinsky et al., 2025). Many everyday tendencies have also been ascribed, in the popular press (DeMelo, 2019), to pre-crastination, such as answering emails too soon, carrying too many groceries in too few trips, submitting manuscript or proposals long before they are due, rushing into surgeries before getting second opinions, and even convicting people too quickly. In general, all of these examples illustrate the desire to clear one’s mind by completing tasks sooner rather than later, perhaps to reduce “psychic tension,” as suggested by Lewin (1935), the founder of social psychology. Noting how widespread pre-crastination is, Wasserman (2019) has suggested that pre-crastination may be a fundamental drive.

Returning to the relation between pre-crastination and cognitive frontloading, it is worth returning to Fig. 1 and emphasizing that the key idea here is that responses called for by multiple stimuli are prepared before the responses are performed. Thus, as seen in Fig. 1, rather than producing responses one at a time following processing of their corresponding stimuli (top panel), the responses are specified *en masse* and then are executed in batch mode (bottom panel). This causes the time before the first response to be lengthened but allows the subsequent inter-response times to be short, as we observed.

Cognitive frontloading exploits delegation of response production to specialized output mechanisms, thereby enabling processing of upcoming stimuli while responses are underway. The number or complexity of response sequences that can be generated *en masse* presumably depends on the capacity of the relevant stores (cf. Logan, 1983). If the capacity were limited to just one stimulus or response, the mode of control would devolve into chaining (top panel Fig. 1). By contrast, if the capacity were larger, the number of stimuli and responses that could be frontloaded would increase and the time to start the sequences would be expected to be independent of the serial position of the uncertain response, as found here, and would be expected to grow with the length of the sequence. Data consistent with that prediction have been reported.^5^

Postulating cognitive frontloading calls to mind cognitive offloading, which has been studied extensively (Gilbert, 2024; Risko & Gilbert, 2016). Whereas cognitive offloading shunts information to external sources or agents for later access, cognitive frontloading translates multiple stimuli into commands for corresponding responses for execution by motor processors. Insofar as perception and action must be coordinated all the time whereas shunting information to external sources for later access may be less common, it is possible that cognitive frontloading occurs more often and is more fundamental than cognitive offloading.

A penultimate remark is that cognitive frontloading is not the same as action planning more generally. Extensive evidence exists for action planning, including anticipatory errors (Dell & Reich, 1980; Lashley, 1951; Norman, 1981), adaptive modification of early actions depending on actions to come (Fowler, 1980; Rosenbaum et al. 2012), and speeding or smoothing of transitions between action elements that fall within the planning span (Meulenbroek et al., 1996; Fischer, Rosenbaum, & Vaughan, 1997; Logan & Crump, 2011). The novel idea regarding cognitive frontloading is that, precisely to ensure that action elements cohere, the system assembles a coherent plan in advance. This policy enables formation of interactive networks of the kind that permit temporally extended performance (Dell, 1986; Meyer & Gordon, 1985).

A final implication of our results is that the way stimuli were processed here was apparently tuned to the way their associated responses would be generated. Although the stimuli (the targets) could have been processed piecemeal, one at a time with each stimulus triggering its response, our participants apparently did not use that strategy. Rather, they completed the bulk of their stimulus-processing and decision-making up front. Cognitive frontloading was applied, therefore, to stimulus processing in the service of response preparation. This conclusion is consistent with embodied-perception views (Witt, 2011) and suggests, for the first time as far as we know, that embodiment affects segmentation of successive stimuli according to how the stimuli will be acted upon.

## Data Availability

All of the data from this experiment and the next ones are available at https://osf.io/uphfj/.

## Footnotes

1 Rosenbaum et al. (2022) noted that this “double-response” task, as they called it, it was a new entry in the set of reaction-time tasks first created by Donders (1868/1969) and extended by others.

2 Our use of the term response specification in this model and the others was meant to allow for visuo-spatial tagging of targets as well as or instead of motor planning per se. The division between visuo-spatial specification and motor planning is actually much harder to operationalize than one might expect (Rosenbaum, 2010, 2022). For present purposes, it was unimportant to break them apart. The more important distinction here is between response specification and response execution. This is a well-known and well-accepted distinction, even granting that there is extensive evidence for the view that the two processes can co-occur; that is, while executing one response, a later response may be specified (e.g., Song & Nakayama, 2009).

3 Our use of the term response specification was meant to allow for visuo-spatial tagging of targets instead of, or in addition, to motor planning per se. The division between visuo-spatial specification and motor planning is largely artificial (Rosenbaum, 2010, 2022). Other models are possible as well, of course. We focused on these four to spotlight slow and fast pre-crastination and procrastination and, in particular, to provide a way of spotlighting the possibility that there could be pre-crastination with the fastest possible start-time. Also, we did not mean to suggest that if the Fast Pre-crastination model were supported, that would mean participants placed a greater premium on minimizing start times than minimizing total targeting times.

4 We are indebted to a reviewer for suggesting that we check for this. Because it was not a prediction we made in advance, we decided to report this result in the General Discussion section.

5 Previous studies have shown that the time to choose between extended response sequences does in fact increase with the length of the sequence, up to a limit, consistent with established estimates of the span on immediate memory. More specifically, the time to start either of two possible sequences grows with the number of responses to be produced (Inhoff et al., 1984; Rosenbaum et al., 1984), where the same stimulus, either an X or an O, designated the sequence to be produced, with the two possible sequences varying over blocks of trials. Participants memorized the two possible sequences per trial, which could have biased the results in favor of an outcome consistent with cognitive frontloading because, with memorization, cognitive frontloading was obligatory, whereas here, cognitive frontloading was optional, making our results potentially more interesting, because the stimuli requiring responses (the targets to be clicked on) were visible throughout the trials.
